# The Value of Left Atrial Strain and Strain Rate for Prediction of Early Postoperative Atrial Fibrillation Following Isolated Off‐Pump Coronary Artery Bypass Grafting in Low‐Risk Patients

**DOI:** 10.1111/anec.70230

**Published:** 2026-08-25

**Authors:** Leila Bigdelu, Ali Azari, Ossama Maadarani, Somaye Bakefayat, Negar Ebrahimi, Maryam Emadzadeh

**Affiliations:** ^1^ Patient Safety Research Center, Clinical Research Institute Mashhad University of Medical Sciences Mashhad Iran; ^2^ Cardiovascular Surgery Department Mashhad University of Medical Sciences Mashhad Iran; ^3^ Critical Care Unit, Internal Medical Department Ahmadi Hospital‐Kuwait Oil Company Al Ahmadi Kuwait; ^4^ Department of Cardiology, Ghaem Hospital Mashhad University of Medical Sciences Mashhad Iran; ^5^ Cardiac Primary Prevention Research Center, Cardiovascular Diseases Research Institute Tehran University of Medical Sciences Tehran Iran; ^6^ Metabolic Syndrome Research Center Mashhad University of Medical Sciences Mashhad Iran; ^7^ Clinical Research Development Unit Ghaem Hospital, Mashhad University of Medical Sciences Mashhad Iran

**Keywords:** atrial fibrillation, left atrium, off‐pump coronary artery bypass, strain

## Abstract

**Background:**

Following coronary artery bypass grafting (CABG) surgery, postoperative atrial fibrillation (POAF) is a prevalent arrhythmia. Prior research has demonstrated a paucity of reliable approaches to predict early POAF after CABG, particularly in low‐risk subjects. The present investigation proposed to assess how effectively left atrial (LA) strain and LA strain rate predict early POAF after isolated off‐pump CABG (OPCAB) in low‐risk individuals.

**Methods:**

One hundred and twenty‐three low‐risk subjects with sinus rhythm who underwent isolated OPCAB were assessed in this prospective research. The velocity vector imaging (VVI) method was utilized to measure LA strain and LA strain rate. Subsequently, the patients were monitored post‐surgery for 1 week to assess whether early POAF had occurred.

**Results:**

In contrast to those who did not experience early POAF, individuals with early POAF showed significantly lower LA strain (21.25 vs. 37.59, *p* < 0.001), lower LA strain rate (1.37 vs. 2.73, *p* < 0.001), and higher left atrial volume index (LAVI) (*p* < 0.001). A 1‐unit increase in LA strain was associated with a 33% decrease in the odds of early POAF (OR = 0.67, 95% CI: 0.50–0.89; *p* = 0.006).

**Conclusion:**

Preoperative left atrial strain measurements may serve as an effective predictor of early POAF in low‐risk patients after isolated OPCAB. Rather than treating all patients undergoing CABG, identifying the group at greater risk for early POAF may enable targeted prophylactic therapy.

## Graphical abstract

Postoperative atrial fibrillation is a common complication following coronary artery bypass grafting and may lead to prolonged hospital stays and increased risk of stroke‐related mortality and morbidity. This study found that lower preoperative left atrial strain and strain rate are effective predictors for POAF in low‐risk patients undergoing CABG.

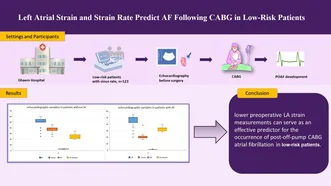

## 1 Introduction

The most frequent arrhythmia following coronary artery bypass grafting (CABG) is postoperative atrial fibrillation (POAF). This arrhythmia leads to an elevated mortality rate and lengthens hospital stays (Seo et al. 2021). POAF may be discovered in 20%–40% of patients undergoing cardiac surgical procedures (Eikelboom et al. 2021). Furthermore, this arrhythmic event has been acknowledged as a predictor of cerebrovascular strokes following CABG and its related morbidities and mortalities (Laimoud et al. 2022). To prevent the risk of postoperative arrhythmia, prophylactic therapy utilizing beta‐adrenergic blockers has been proven to be effective and is frequently prescribed both before and after cardiac surgery (Khan et al. 2013).

Various underlying risk factors, including left ventricular hypertrophy, COPD, metabolic syndrome, and obesity, have been attributed to POAF (Seo et al. 2021; Zacharias et al. 2005; Auer et al. 2005; Echahidi et al. 2007; Lopes and Agrawal 2022; Higgs et al. 2020). Intraoperative risk profiles, including atrial injury or ischemia, vascular catheter insertion, abrupt changes in the volume of circulating blood, and operation using cardiopulmonary bypass, have also received attention. Moreover, previous evidence has revealed that functional and structural alterations in the left atrium (LA) contribute to the development and recurrence of atrial fibrillation (AF) as well as localized disruption of electrical conduction (Parsaee et al. 2014). Echocardiographic measurements of LA, including LA size, volume, and function (identified by strain), are valuable tools for recognizing patients with heightened risk of POAF (Reddy Y et al. 2010; Candan et al. 2013).

LA strain is usually measured using 2‐dimensional speckle tracking echocardiography (2D STE), tissue Doppler imaging (TDI), and vector velocity imaging (VVI) (Alimi et al. 2022). VVI is an endocardial border tracking method that remains particularly effective in tracking the movement of structures with thin walls, including the LA. Strain rate imaging can provide valuable insights into assessing compromised atrial contractility, especially in patients with AF. By analyzing data on LA strain rate, it is possible to noninvasively assess atrial reverse remodeling in patients with AF (Her et al. 2013).

Although it has been shown that LA strain is associated with POAF, most available evidence has been derived from heterogeneous cardiac surgery populations, or on‐pump CABG (Misra et al. 2026). Cardiopulmonary bypass induces a substantial inflammatory response that contributes to POAF (Maesen et al. 2012), and on‐pump CABG, compared with off‐pump CABG (OPCAB), is linked to a higher inflammatory response (Mirhafez et al. 2021). Therefore, by selecting low‐risk participants undergoing isolated OPCAB, we sought to minimize the influence of conventional risk factors and cardiopulmonary bypass‐related inflammation. This approach allowed us to more specifically evaluate whether LA strain and LA strain rate may independently predict early POAF. Accordingly, the goal of this study was to evaluate the predictive significance of LA strain and LA strain rate for early POAF after isolated OPCAB in low‐risk patients.

## 2 Methods and Materials

### 2.1 Study Setting and Population

In this prospective research, low‐risk individuals with sinus rhythm who were scheduled to undergo isolated OPCAB were enrolled at Ghaem Hospital, Mashhad University of Medical Sciences, Mashhad, Iran.

Low risk was defined as individuals who had only one or no major cardiovascular risk factors, including age ≥ 65, diabetes mellitus, hypertension, history of transient ischemic attack (TIA) or stroke, heart failure, or vascular problems. Individuals who had already experienced AF, chronic lung disease, pulmonary hypertension, or a history of taking anti‐arrhythmic agents were excluded. Demographic and echocardiographic data were gathered for all the individuals recruited in this investigation.

### 2.2 Echocardiographic Evaluation

One day before CABG surgery, transthoracic echocardiography (TTE) was performed using a Siemens ACUSON SC2000 ultrasound system equipped with a 4V1c transducer (with frequency bandwidth of 1.25–4.5 MHz). Individuals were in the left lateral decubitus position while imaging was conducted.

An echocardiologist followed standard techniques consistent with the latest guidelines of the American Society of Echocardiography (Lang et al. 2015). M‐mode, two‐dimensional, and Doppler measurements were attained.

Left ventricular end‐diastolic and end‐systolic volumes were assessed, and left ventricular ejection fraction (LVEF) was calculated utilizing the Simpson method in the apical 4‐chamber view. Left atrial volume index (LAVI) was measured by a biplane method of discs evaluating LA volume indexed for body surface area. An LAVI value of 34 mL/m^2^ or higher was considered indicative of LA enlargement for both sexes (Lazzeroni et al. 2016).

### 2.3 Left Atrial Strain Analysis

LA strain was measured by the VVI technique. This technique employs endocardial border tracking to accurately estimate the movement of structures with thin walls like LA (Alimi et al. 2022). Evaluation of LA strain enables a thorough assessment of reservoir, conduit, and contractile performance (Gan et al. 2018). During the reservoir phase, peak LA longitudinal strain was examined.

**FIGURE 1:**
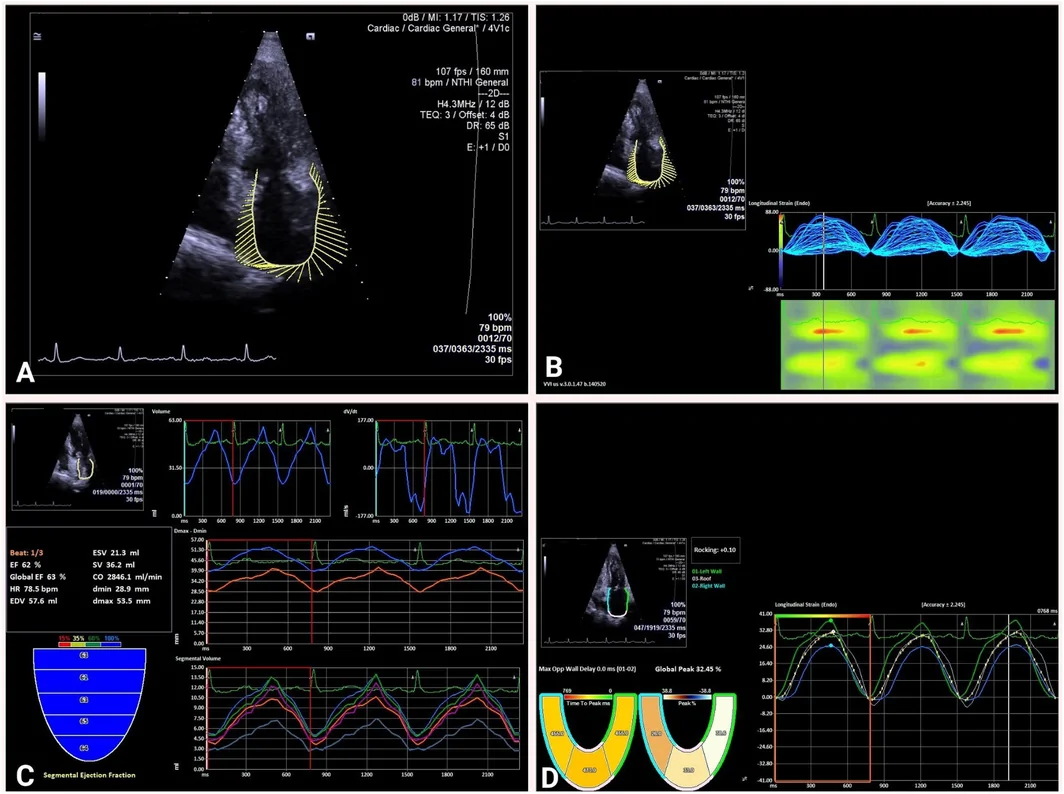
VVI for left atrial strain and left atrial strain rate: (A) VVI tracings of the left atrial endocardial border from the apical four‐chamber view; (B) Longitudinal left atrial strain curve; (C) Segmental strain, volume, and ejection fraction curves in 3 consecutive cardiac cycles and other myocardial deformation parameters; (D) Longitudinal left atrial strain curve and global peak.

To perform the VVI method, 2‐dimensional pictures of the apical 2‐ and 4‐chamber views were acquired at frame rates ranging from 70 to 100 frames/s. Moreover, to calculate strain, we employed R R gating. The LA's endocardium was manually traced. The region of interest (ROI) was defined from the lateral to the medial aspect of the mitral annulus, while the pulmonary veins and LA appendage were not included. Measures of LA strain and LA strain rate were computed automatically (Figure 1).

### 2.4 Follow‐Up and ECG Analysis

Following surgery, all subjects were constantly monitored in the ICU and subsequent hospitalization using a central monitoring system. Individuals were monitored for a week in order to look into the occurrence of POAF. Until discharge, 12‐lead ECG recordings were also performed daily. Further ECG recordings were performed if new signs or symptoms, including palpitations, tachycardia, or an irregular pulse, developed. The endpoint of the investigation was defined as the no visible P waves and the presence of irregular R‐R intervals on rhythm strips or 12‐lead electrocardiographic recordings. AF episodes that lasted more than 5 min were counted (Mitchell et al. 2005; Gaudino et al. 2023).

### 2.5 Ethical Approval

All individuals enrolled in the study completed informed consent forms. Participants' data were extracted while considering the ethical principles. This study acquired ethical approval from the Organizational Ethics Committee of the Faculty/Region of Mashhad University of Medical Sciences. The ethical approval code was IR.MUMS.MEDICAL.REC.1393.765.

### 2.6 Statistical Analysis and Report

Quantitative variables were presented as mean ± standard deviation (SD), and qualitative variables were reported using absolute frequencies and percentages. In order to assess the normality of data, the Kolmogorov–Smirnov test was utilized. The χ2 test or Fisher's exact test was implemented for qualitative variables when there were more than 20% of cells with an expected count of less than five. Continuous variables were compared between groups with POAF and without POAF using the Mann–Whitney *U* test because data were not normally distributed. The correlation between quantitative indices was assessed using Pearson's or Spearman's tests, as appropriate. Independent predictors of early POAF were detected using multivariable logistic regression analysis. SPSS software version 16.0 for Windows (SPSS Inc., Chicago, IL) was utilized for the statistical analysis. *p* < 0.05 was considered to be statistically significant.

## 3 Results

This study included 123 patients (50.4% male) who had OPCAB. Regarding cardiovascular risk factors, 35.8% were diabetic, and 43.9% were hypertensive. The mean serum creatinine level was 1.14 ± 0.19 mg/dL (ranging from 0.8 to 1.8 mg/dL). Additionally, the mean LVEF measured by echocardiography was 50.25% ± 7.32% (range: 20%–60%) while the mean strain was 35.59 ± 6.75 (range: 16.3–45.6), the mean strain rate was 2.56 ± 0.60 (range: 1.0–3.6), and the mean LAVI was 25.58 ± 4.62 mL/m^2^ (range: 19–40 mL/m^2^).

POAF was detected in 15 patients with an overall incidence of 12.2%. Individuals with POAF were older and had a significantly lower LVEF in comparison with those without this arrhythmia (*p* < 0.001, *p* = 0.001, respectively). There were no significant variations between the with and without POAF subjects regarding sex (46.7% male versus 50.9% male, *p* = 0.757), diabetes mellitus (26.7% vs. 37.0%, *p* = 0.432), and hypertension (40.0% versus 44.4%, *p* = 0.745). Moreover, there was no significant difference in mean serum creatinine level among both groups with and without POAF (1.14 ± 0.19 versus 1.14 ± 0.20, *p* = 0.949) (Table 1).

**TABLE 1:** Comparison of baseline and echocardiographic characteristics in the groups with and without atrial fibrillation.

Item*	Group with AF (*n* = 15)	Group without AF (*n* = 108)	*p* *
Male sex	7 (46.7)	55 (50.9)	0.757
Age (years)	65.67 ± 3.69	58.94 ± 5.56	**< 0.001**
History of diabetes mellitus	4 (26.7)	40 (37.0)	0.432
History of hypertension	6 (40.0)	48 (44.4)	0.745
Serum creatinine (mg/dL)	1.14 ± 0.20	1.14 ± 0.19	0.949
LA strain (%)	21.25 ± 5.23	37.59 ± 3.93	**< 0.001**
LA Strain rate (s^−1^)	1.37 ± 0.41	2.73 ± 0.41	**< 0.001**
LA volume index (mL/m^2^)	32.60 ± 3.56	24.60 ± 3.86	**< 0.001**
LVEF (%)	44.60 ± 11.16	51.04 ± 6.30	**0.001**

* Bold values indicate statistically significant results.

**FIGURE 2:**
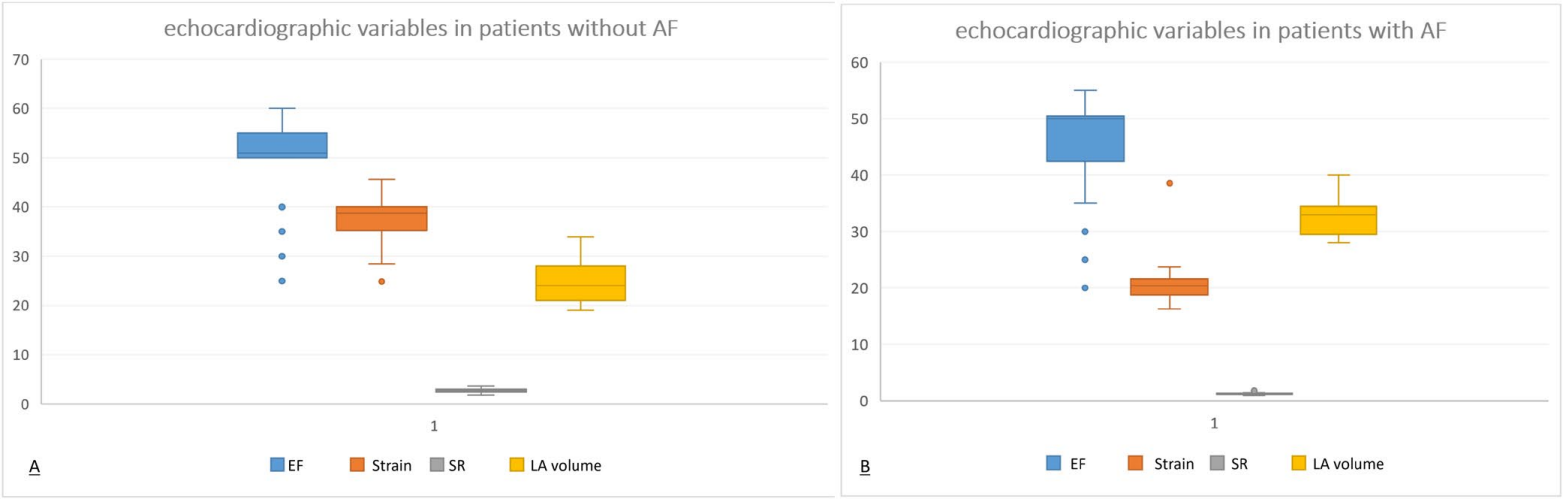
Boxplots of echocardiographic variables in patients. (A) Echocardiographic variables in individuals without atrial fibrillation, (B) Echocardiographic variables in individuals with atrial fibrillation. EF, ejection fraction; SR, strain rate.

Based on our analysis, patients with POAF had significantly lower LA strain (21.25 vs. 37.59, *p* < 0.001), lower strain rate (1.37 vs. 2.73, *p* < 0.001), and higher LAVI (*p* < 0.001) when compared to patients without POAF (Table 1 and Figure 2).

Correlation analysis showed that both LA strain and LA strain rate were negatively correlated with age (*r* = −0.359 and *r* = −0.345, respectively; both *p* < 0.001); however, LAVI was positively correlated with age (*r* = 0.319, *p* < 0.001) (Table 2).

**TABLE 2:** Correlation matrix of clinical and echocardiographic variables.

		Age	Creatinine	LVEF	Strain	Strain rate	LA volume
Age	Pearson Correlation	1	−0.127	−0.224	−0.359	−0.345	0.319
	*p* Value*		0.162	**0.013**	**< 0.001**	**< 0.001**	**< 0.001**
Creatinine	Pearson Correlation	—	1	0.133	0.042	0.031	−0.066
	*p* Value	—		0.142	0.641	0.730	0.466
LVEF	Pearson Correlation	—	—	1	0.527	0.527	−0.566
	*p* Value	—	—		**< 0.001**	**< 0.001**	**< 0.001**
Strain	Pearson Correlation	—	—	—	1	0.967	−0.748
	*p* Value	—	—	—		**< 0.001**	**< 0.001**
Strain rate	Pearson Correlation	—	—	—	—	1	−0.751
	*p* Value	—	—	—	—		**< 0.001**
LA volume	Pearson Correlation	—	—	—	—	—	1
	*p* Value	—	—	—	—	—	

* Bold values indicate statistically significant results.

Multivariate logistic regression analysis demonstrated that LA strain was the only independent predictor of POAF (OR = 0.67, 95% CI: 0.50–0.89; *p* = 0.006). The analysis indicates that each 1‐unit increase in LA strain is associated with a 33% reduction in the odds of developing POAF (Table 3).

**TABLE 3:** Multivariable logistic regression analysis of predictors of early postoperative atrial fibrillation.

Variable	Odds ratio	95% confidence interval	*p*
Age	2.01	0.92–3.36	0.07
Left atrial strain (%)	0.67	0.50–0.89	0.006
Left atrial volume index (mL/m^2^)	1.70	0.77–3.73	0.18

## 4 Discussion

We designed this prospective investigation in order to examine the predictive significance of LA strain and LA strain rate for early POAF after isolated OPCAB in low‐risk subjects. Our study demonstrated that individuals who experienced early POAF had significantly lower strain and strain rate and higher LAVI than those who did not. Nevertheless, only LA strain remained an independent predictor of early POAF, following multivariable adjustment.

In general populations, the incidence of AF is about 1.8% (Schnabel et al. 2012; Teh et al. 2023). For those under 70 years old, the rate is only 0.4%, while it increases to 2%–4% for individuals over 70 and up to 18% for those aged 80 years and above (Teh et al. 2023; Norberg et al. 2013). This frequency is about 5% in subjects undergoing general surgical procedures (Alqahtani 2010). Among the hospitalized population, this frequency ranges from 8% to 14% (Shi et al. 2002). The reported incidence of AF in patients undergoing open cardiac operations is much greater, varying between 3.1% and 91%, with large‐scale studies suggesting a rate of approximately 30% (Ellis 2013; Kawczynski et al. 2021). In our study, which focused on low‐risk patients, an average incidence of 12.1% was detected for early POAF, which is lower than that described in earlier research. Our selection of low‐risk patients may partly explain this disparity. By minimizing the impact of conventional risk factors, this approach enabled a more focused assessment of the contribution of left atrial function to the early POAF development. The other possible explanation is the short follow‐up period, which may have contributed to the underestimation of the true incidence.

Previous literature has identified that increasing age and enlargement of the left atrium were independent predictors of POAF (Wang et al. 2012; Nakai et al. 2002; Somesarayi et al. 2023). The incidence of AF varies with age, with an OR of 1.60 per decade of additional life, ranging from 13% for subjects under 50 to 52% for those over 80. This finding aligns with prior investigations indicating POAF is reliably predicted by older age (Her et al. 2013; Schnabel et al. 2012; Norberg et al. 2013). The heart may undergo structural alterations as we age, including increased chamber size and fibrosis (Di Salvo et al. 2005; Verdejo et al. 2016). In our study, subjects who experienced early POAF were significantly older than those who did not. However, age was not an independent predictor of early POAF after conducting multivariable logistic analysis. This finding may be caused by the relatively homogeneous, low‐risk nature of our study population and the limited number of POAF events. Moreover, in line with some previous reports, we did not find any significant differences between the subjects with and without POAF regarding sex (Hashemzadeh et al. 2013; Abdelrazek et al. 2021).

According to various studies, POAF tends to happen in the first 5 days after surgery, peaking on the 2nd post‐operative day (Parsaee et al. 2014; Zhang et al. 2023). Although the etiology of POAF remains uncertain (Zhang et al. 2023; Tayyareci et al. 2010), numerous factors, including surgical trauma, insufficient cooling of the atrium, inadequate preservation of atrial tissue by cardioplegia, post‐operative pericarditis, elevated sympathetic function, euthyroid sick syndrome, and withdrawal from beta‐blockers, have been suggested as potential contributors (Shi et al. 2002; Ellis 2013; Di Salvo et al. 2005). The hypothesis of “dispersion of refractoriness” appears to be a reasonable description, presuming that the susceptibility of atrial tissue to the development of AF is caused by dispersion or deficiency in conformity of atrial refractory periods as the underlying mechanism (van Staveren and de Groot 2020).

LA size is a widely employed parameter to investigate structural changes in LA. Research has elaborated that development of AF in the general population can be predicted by LA size (including diameter and volume) (Haffajee et al. 2011). Due to the asymmetry and enlargement of the chamber, volume has been considered a more accurate evaluation of left atrial size compared to diameter (Tsang et al. 2006). Osranek et al. (2006) evaluated patients who underwent CABG and concluded LAVI greater than 32 mL/m^2^ was an indicator of POAF. Regardless of age and other clinical parameters, individuals with LAVI above this threshold had a five‐fold higher probability of POAF occurrence. In accordance with previous studies, our research also indicated larger LAVI values were detected in patients with POAF (*p* < 0.01). However, after conducting multivariable logistic regression, LAVI did not remain an independent predictor. In contrast, strain of LA remained as an independent predictor, which may suggest that impaired atrial function precedes structural remodeling in low‐risk patients; yet, this interpretation should be made with caution.

Regional deformation of LA walls and global LA function can be noninvasively evaluated by TDI, VVI, and 2D STE (Vieira et al. 2014; Mor‐Avi et al. 2011; Motoki et al. 2012). Previous studies have demonstrated that preoperative LA strain can predict POAF in adult patients who undergo cardiac surgery (Misra et al. 2026). Tayyareci et al. (2010) showed that early diastolic strain rate, systolic strain rates, and LA strain assessed by VVI were independent markers to predict POAF in patients undergoing CABG. Similarly, Gabrielli et al. (2011) reported that LA strain and LA strain rate measured by STE were lower in this population. Likewise, in patients undergoing mitral valve surgery, LA longitudinal strain can predict the occurrence of POAF. This parameter could potentially facilitate risk stratification by detecting individuals at greater risk of POAF. Moreover, it will guide cardiologists in selecting appropriate intensive prophylactic anti‐arrhythmic therapy (Candan et al. 2013).

It has been suggested that pre‐operative LA dysfunction is an important factor in developing POAF through neurohumoral activation and enhanced atrial electrical vulnerability (Tayyareci et al. 2010). Cameli et al. (2012) reported that global positive strain is an independent predictor of cardiovascular events, beyond the standard factors commonly used for LA investigation. In our study, by restricting our participants to low‐risk patients undergoing isolated OPCAB, we sought to minimize the influence of conventional risk factors and cardiopulmonary bypass‐related inflammation. This approach allowed us to more specifically determine whether impaired LA function could predict early POAF.

Different studies in subjects with AF have highlighted that LA strain predicts success in sinus rhythm restoration following ablation or electrical cardioversion. Additionally, it predicts the likelihood of recurrence of AF following successful cardioversion (Thomas et al. 2007; Kuppahally et al. 2010). Azemi et al. (2012) revealed that decreased LA strain could serve as a sensitive marker for elevated risk of stroke or TIA in individuals with low‐risk CHADS2 scores, AF, and a history of TIA or stroke. These outcomes indicate that strain may be a beneficial measure for decision‐making regarding the prescription of oral anticoagulation in this population.

There may be some possible limitations in this study. First, our work was carried out at a single center, and the sample size was relatively small. This limitation may restrict the external validity of our results. Secondly, the low number of POAF events may have limited the statistical power and stability of our multivariable logistic regression model, resulting in relatively wide confidence intervals for some variables. Hence, the predictive performance of the model should be interpreted cautiously and requires external validation in larger cohorts. Thirdly, given that most POAF occurs within the early postoperative period, our patients were followed for 1 week after surgery; however, some episodes of POAF may have occurred after this period, which results in POAF underestimation. Future studies with a larger sample size and longer follow‐up are warranted to validate the results of our investigation in low‐risk subjects undergoing isolated OPCAB.

## 5 Conclusion

Preoperative LA strain measurements may serve as an independent predictor of early POAF in low‐risk subjects after isolated OPCAB. Identifying the group at elevated risk of early POAF may enable targeted prophylactic treatment rather than targeting all patients undergoing OPCAB. Future research with a prolonged follow‐up and a larger study population is required to further evaluate our findings in low‐risk patients undergoing isolated OPCAB.

## Author Contributions

L.B. and S.B. conceived and designed the study. L.B., S.B., and A.A. collected the data. L.B., S.B., N.E., and A.A. conducted the literature search. M.E., S.B., and N.E. analyzed the data and performed the statistical analysis. L.B., S.B., M.E., N.E., and O.M. interpreted the data. L.B., S.B., O.M., and N.E. drafted the article. All the authors critically revised this article and approved the final version of the manuscript.

## Funding

The authors have nothing to report.

## Ethics Statement

This study fully meets the terms of the Declaration of Helsinki. The study acquired ethical approval from the Organizational Ethics Committee of the Faculty/Region of Mashhad University of Medical Sciences. The ethical approval code was IR.MUMS.MEDICAL.REC.1393.765.

## Consent

All individuals enrolled in the study completed informed consent forms.

## Conflicts of Interest

The authors declare no conflicts of interest.

## Data Availability

The data that support the findings of this study are available on request from the corresponding author. The data are not publicly available due to privacy or ethical restrictions.

## References

[anec70230-bib-0001] Abdelrazek, G. , K. Mandour , M. Osama , and K. Elkhashab . 2021. “Strain and Strain Rate Echocardiographic Imaging Predict Occurrence of Atrial Fibrillation in Post‐Coronary Artery Bypass Grafting Patients.” Egyptian Heart Journal 73, no. 1: 62. 10.1186/s43044-021-00188-z PMC8254834 34216305

[anec70230-bib-0002] Alimi, H. , L. Bigdelu , H. Poorzand , et al. 2022. “Assessment of Functional and Structural Echocardiography Parameters in Patients With Frequent Premature Ventricular Contractions Without Structural Heart Disease.” ARYA Atherosclerosis 18, no. 2: 1–7. 10.48305/arya.2022.16310 PMC9931611 36819835

[anec70230-bib-0003] Alqahtani, A. A. 2010. “Atrial Fibrillation Post Cardiac Surgery Trends Toward Management.” Heart Views 11, no. 2: 57. 21187998 10.4103/1995-705X.73212 PMC3000913

[anec70230-bib-0004] Auer, J. , T. Weber , R. Berent , C. K. Ng , G. Lamm , and B. Eber . 2005. “Risk Factors of Postoperative Atrial Fibrillation After Cardiac Surgery.” Journal of Cardiac Surgery 20, no. 5: 425–431. 16153272 10.1111/j.1540-8191.2005.2004123.x

[anec70230-bib-0005] Azemi, T. , V. M. Rabdiya , S. R. Ayirala , L. D. McCullough , and D. I. Silverman . 2012. “Left Atrial Strain Is Reduced in Patients With Atrial Fibrillation, Stroke or TIA, and Low Risk CHADS 2 Scores.” Journal of the American Society of Echocardiography 25, no. 12: 1327–1332. 23067897 10.1016/j.echo.2012.09.004

[anec70230-bib-0006] Cameli, M. , M. Lisi , M. Focardi , et al. 2012. “Left Atrial Deformation Analysis by Speckle Tracking Echocardiography for Prediction of Cardiovascular Outcomes.” American Journal of Cardiology 110, no. 2: 264–269. 22497676 10.1016/j.amjcard.2012.03.022

[anec70230-bib-0007] Candan, O. , N. Ozdemir , S. M. Aung , et al. 2013. “Left Atrial Longitudinal Strain Parameters Predict Postoperative Persistent Atrial Fibrillation Following Mitral Valve Surgery: A Speckle Tracking Echocardiography Study.” Echocardiography 30, no. 9: 1061–1068. 23600893 10.1111/echo.12222

[anec70230-bib-0008] Di Salvo, G. , P. Caso , R. L. Piccolo , et al. 2005. “Atrial Myocardial Deformation Properties Predict Maintenance of Sinus Rhythm After External Cardioversion of Recent‐Onset Lone Atrial Fibrillation a Color Doppler Myocardial Imaging and Transthoracic and Transesophageal Echocardiographic Study.” Circulation 112, no. 3: 387–395. 16006491 10.1161/CIRCULATIONAHA.104.463125

[anec70230-bib-0009] Echahidi, N. , D. Mohty , P. Pibarot , et al. 2007. “Obesity and Metabolic Syndrome Are Independent Risk Factors for Atrial Fibrillation After Coronary Artery Bypass Graft Surgery.” Circulation 116, no. 11_supplement: I‐213–I‐219. 10.1161/CIRCULATIONAHA.106.681304 17846306

[anec70230-bib-0010] Eikelboom, R. , R. Sanjanwala , M.‐L. Le , M. H. Yamashita , and R. C. Arora . 2021. “Postoperative Atrial Fibrillation After Cardiac Surgery: A Systematic Review and Meta‐Analysis.” Annals of Thoracic Surgery 111, no. 2: 544–554. 32687821 10.1016/j.athoracsur.2020.05.104

[anec70230-bib-0011] Ellis, S. G. 2013. “Coronary Revascularization for Patients With Diabetes: Updated Data Favor Coronary Artery Bypass Grafting.” Journal of the American College of Cardiology 61, no. 8: 817–819. 23428215 10.1016/j.jacc.2012.12.003

[anec70230-bib-0012] Gabrielli, L. , R. Corbalan , S. Córdova , et al. 2011. “Left Atrial Dysfunction Is a Predictor of Postcoronary Artery Bypass Atrial Fibrillation: Association of Left Atrial Strain and Strain Rate Assessed by Speckle Tracking.” Echocardiography 28, no. 10: 1104–1108. 21967453 10.1111/j.1540-8175.2011.01518.x

[anec70230-bib-0013] Gan, G. C. , A. Ferkh , A. Boyd , and L. Thomas . 2018. “Left Atrial Function: Evaluation by Strain Analysis.” Cardiovascular Diagnosis and Therapy 8, no. 1: 29–46. 29541609 10.21037/cdt.2017.06.08 PMC5835645

[anec70230-bib-0014] Gaudino, M. , A. Di Franco , L. Q. Rong , J. Piccini , and M. Mack . 2023. “Postoperative Atrial Fibrillation: From Mechanisms to Treatment.” European Heart Journal 44, no. 12: 1020–1039. 36721960 10.1093/eurheartj/ehad019 PMC10226752

[anec70230-bib-0015] Haffajee, J. A. , Y. Lee , A. A. Alsheikh‐Ali , J. T. Kuvin , N. G. Pandian , and A. R. Patel . 2011. “Pre‐Operative Left Atrial Mechanical Function Predicts Risk of Atrial Fibrillation Following Cardiac Surgery.” JACC: Cardiovascular Imaging 4, no. 8: 833–840. 21835375 10.1016/j.jcmg.2011.03.019

[anec70230-bib-0016] Hashemzadeh, K. , M. Dehdilani , and M. Dehdilani . 2013. “Postoperative Atrial Fibrillation Following Open Cardiac Surgery: Predisposing Factors and Complications.” Journal of Cardiovascular and Thoracic Research 5, no. 3: 101–107. 24252985 10.5681/jcvtr.2013.022 PMC3825396

[anec70230-bib-0017] Her, A.‐Y. , J.‐Y. Kim , Y. H. Kim , et al. 2013. “Left Atrial Strain Assessed by Speckle Tracking Imaging Is Related to New‐Onset Atrial Fibrillation After Coronary Artery Bypass Grafting.” Canadian Journal of Cardiology 29, no. 3: 377–383. 22902158 10.1016/j.cjca.2012.06.006

[anec70230-bib-0018] Higgs, M. , J. Sim , and V. Traynor . 2020. “Incidence and Risk Factors for New‐Onset Atrial Fibrillation Following Coronary Artery Bypass Grafting: A Systematic Review and Meta‐Analysis.” Intensive & Critical Care Nursing 60: 102897. 32601010 10.1016/j.iccn.2020.102897

[anec70230-bib-0019] Kawczynski, M. J. , M. Gilbers , S. Van De Walle , et al. 2021. “Role of Pre‐Operative Transthoracic Echocardiography in Predicting Post‐Operative Atrial Fibrillation After Cardiac Surgery: A Systematic Review of the Literature and Meta‐Analysis.” EP Europace 23, no. 11: 1731–1743. 34000038 10.1093/europace/euab095

[anec70230-bib-0020] Khan, M. F. , C. S. Wendel , and M. R. Movahed . 2013. “Prevention of Post–Coronary Artery Bypass Grafting (CABG) Atrial Fibrillation: Efficacy of Prophylactic Beta‐Blockers in the Modern Era.” Annals of Noninvasive Electrocardiology 18, no. 1: 58–68. 23347027 10.1111/anec.12004 PMC6932325

[anec70230-bib-0021] Kuppahally, S. S. , N. Akoum , N. S. Burgon , et al. 2010. “Left Atrial Strain and Strain Rate in Patients With Paroxysmal and Persistent Atrial Fibrillation Relationship to Left Atrial Structural Remodeling Detected by Delayed‐Enhancement MRI.” Circulation: Cardiovascular Imaging 3, no. 3: 231–239. 20133512 10.1161/CIRCIMAGING.109.865683

[anec70230-bib-0022] Laimoud, M. , M. Maghirang , M. Alanazi , et al. 2022. “Predictors and Clinical Outcomes of Post‐Coronary Artery Bypass Grafting Cerebrovascular Strokes.” Egyptian Heart Journal 74, no. 1: 76. 10.1186/s43044-022-00315-4 PMC9579243 36255549

[anec70230-bib-0023] Lang, R. M. , L. P. Badano , V. Mor‐Avi , et al. 2015. “Recommendations for Cardiac Chamber Quantification by Echocardiography in Adults: An Update From the American Society of Echocardiography and the European Association of Cardiovascular Imaging.” European Heart Journal Cardiovascular Imaging 16, no. 3: 233–271. 25712077 10.1093/ehjci/jev014

[anec70230-bib-0024] Lazzeroni, D. , N. Gaibazzi , M. Bini , et al. 2016. “Prognostic Value of New Left Atrial Volume Index Severity Partition Cutoffs After Cardiac Rehabilitation Program in Patients Undergoing Cardiac Surgery.” Cardiovascular Ultrasound 14, no. 1: 35. 27552988 10.1186/s12947-016-0077-0 PMC4994378

[anec70230-bib-0025] Lopes, L. A. , and D. K. Agrawal . 2022. “Post‐Operative Atrial Fibrillation: Current Treatments and Etiologies for a Persistent Surgical Complication.” Journal of Surgical Research 5, no. 1: 159–172. 10.26502/jsr.10020209 PMC9017863 35445200

[anec70230-bib-0026] Maesen, B. , J. Nijs , J. Maessen , M. Allessie , and U. Schotten . 2012. “Post‐Operative Atrial Fibrillation: A Maze of Mechanisms.” Europace: European Pacing, Arrhythmias, and Cardiac Electrophysiology: Journal of the Working Groups on Cardiac Pacing, Arrhythmias, and Cardiac Cellular Electrophysiology of the European Society of Cardiology 14, no. 2: 159–174. 21821851 10.1093/europace/eur208 PMC3262403

[anec70230-bib-0027] Mirhafez, S. R. , S. H. Khadem , A. Sahebkar , et al. 2021. “Comparative Effects of On‐Pump Versus Off‐Pump Coronary Artery Bypass Grafting Surgery on Serum Cytokine and Chemokine Levels.” IUBMB Life 73, no. 12: 1423–1431. 34601812 10.1002/iub.2566

[anec70230-bib-0028] Misra, S. , D. Das , T. K. Patra , D. P. Borde , and A. Srinivasan . 2026. “Predictive Value of Preoperative Left Atrial Strain Parameters on Postoperative Atrial Fibrillation in Adults Undergoing Cardiac Surgery: A Systematic Review and Meta‐Analysis.” Interdisciplinary CardioVascular and Thoracic Surgery 41, no. 2: ivag035. 41688885 10.1093/icvts/ivag035 PMC12920041

[anec70230-bib-0029] Mitchell, L. B. , D. V. Exner , D. G. Wyse , et al. 2005. “Prophylactic Oral Amiodarone for the Prevention of Arrhythmias That Begin Early After Revascularization, Valve Replacement, or RepairPAPABEAR: A Randomized Controlled Trial.” Journal of the American Medical Association 294, no. 24: 3093–3100. 16380589 10.1001/jama.294.24.3093

[anec70230-bib-0030] Mor‐Avi, V. , R. M. Lang , L. P. Badano , et al. 2011. “Current and Evolving Echocardiographic Techniques for the Quantitative Evaluation of Cardiac Mechanics: ASE/EAE Consensus Statement on Methodology and Indications Endorsed by the Japanese Society of Echocardiography.” European Journal of Echocardiography 12, no. 3: 167–205. 21385887 10.1093/ejechocard/jer021

[anec70230-bib-0031] Motoki, H. , A. Dahiya , M. Bhargava , et al. 2012. “Assessment of Left Atrial Mechanics in Patients With Atrial Fibrillation: Comparison Between Two‐Dimensional Speckle‐Based Strain and Velocity Vector Imaging.” Journal of the American Society of Echocardiography 25, no. 4: 428–435. 22265458 10.1016/j.echo.2011.12.020

[anec70230-bib-0032] Nakai, T. , R. J. Lee , N. B. Schiller , et al. 2002. “The Relative Importance of Left Atrial Function Versus Dimension in Predicting Atrial Fibrillation After Coronary Artery Bypass Graft Surgery.” American Heart Journal 143, no. 1: 181–186. 11773931 10.1067/mhj.2002.120294

[anec70230-bib-0033] Norberg, J. , S. Bäckström , J.‐H. Jansson , and L. Johansson . 2013. “Estimating the Prevalence of Atrial Fibrillation in a General Population Using Validated Electronic Health Data.” Clinical Epidemiology 5, no. 1: 475–481. 24353441 10.2147/CLEP.S53420 PMC3862395

[anec70230-bib-0034] Osranek, M. , K. Fatema , F. Qaddoura , et al. 2006. “Left Atrial Volume Predicts the Risk of Atrial Fibrillation After Cardiac Surgery: A Prospective Study.” Journal of the American College of Cardiology 48, no. 4: 779–786. 16904549 10.1016/j.jacc.2006.03.054

[anec70230-bib-0035] Parsaee, M. , B. Moradi , M. D. F. Maryam Esmaeilzadeh , M. Haghjoo , and H. Bakhshandeh . 2014. “New Onset Atrial Fibrillation After Coronary Artery Bypasses Grafting; an Evaluation of Mechanical Left Atrial Function.” Archives of Iranian Medicine 17, no. 7: 501. 24979564

[anec70230-bib-0036] Reddy Y, M. , R. Satpathy , X. Shen , et al. 2010. “Left Atrial Volume and Post‐Operative Atrial Fibrillation After Aortic Valve Replacement.” Journal of Atrial Fibrillation 3, no. 4: 338. 28496682 10.4022/jafib.338 PMC5398824

[anec70230-bib-0037] Schnabel, R. B. , S. Wilde , P. S. Wild , T. Munzel , and S. Blankenberg . 2012. “Atrial Fibrillation: Its Prevalence and Risk Factor Profile in the German General Population.” Deutsches Ärzteblatt International 109: 293–299. 22577476 10.3238/arztebl.2012.0293 PMC3349225

[anec70230-bib-0038] Seo, E. J. , J. Hong , H.‐J. Lee , and Y.‐J. Son . 2021. “Perioperative Risk Factors for New‐Onset Postoperative Atrial Fibrillation After Coronary Artery Bypass Grafting: A Systematic Review.” BMC Cardiovascular Disorders 21, no. 1: 1–11. 34479482 10.1186/s12872-021-02224-x PMC8414730

[anec70230-bib-0039] Shi, Y. , D. Li , J.‐C. Tardif , and S. Nattel . 2002. “Enalapril Effects on Atrial Remodeling and Atrial Fibrillation in Experimental Congestive Heart Failure.” Cardiovascular Research 54, no. 2: 456–461. 12062350 10.1016/s0008-6363(02)00243-2

[anec70230-bib-0040] Somesarayi, S. A. B. , L. Faridi , K. Mohammadi , et al. 2023. “Conventional and Two‐Dimensional Strain Echocardiography in Predicting Postoperative Atrial Fibrillation After Coronary Artery Bypass Grafting Surgery.” Caspian Journal of Internal Medicine 14, no. 1: 60. 36741482 10.22088/cjim.14.1.60 PMC9878911

[anec70230-bib-0041] Tayyareci, Y. , O. Yildirimtürk , V. Aytekin , K. Memic , F. Behramoglu , and S. Aytekin . 2010. “Preoperative Left Atrial Mechanical Dysfunction Predicts Postoperative Atrial Fibrillation After Coronary Artery Bypass Graft Operation—A Velocity Vector Imaging‐Based Study.” Circulation Journal 74, no. 10: 2109–2117. 20818132 10.1253/circj.cj-10-0197

[anec70230-bib-0042] Teh, R. , N. Kerse , A. Pillai , et al. 2023. “Atrial Fibrillation Incidence and Outcomes in Two Cohorts of Octogenarians: LiLACS NZ.” BMC Geriatrics 23, no. 1: 1–11. 36997900 10.1186/s12877-023-03902-5 PMC10064671

[anec70230-bib-0043] Thomas, L. , T. Mckay , K. Byth , and T. H. Marwick . 2007. “Abnormalities of Left Atrial Function After Cardioversion: An Atrial Strain Rate Study.” Heart 93, no. 1: 89–95. 16818487 10.1136/hrt.2006.088609 PMC1861320

[anec70230-bib-0044] Tsang, T. S. , W. P. Abhayaratna , M. E. Barnes , et al. 2006. “Prediction of Cardiovascular Outcomes With Left Atrial Size: Is Volume Superior to Area or Diameter?” Journal of the American College of Cardiology 47, no. 5: 1018–1023. 16516087 10.1016/j.jacc.2005.08.077

[anec70230-bib-0045] van Staveren, L. N. , and N. M. de Groot . 2020. “Exploring Refractoriness as an Adjunctive Electrical Biomarker for Staging of Atrial Fibrillation.” Journal of the American Heart Association 9, no. 23: e018427. 33238778 10.1161/JAHA.120.018427 PMC7763798

[anec70230-bib-0046] Verdejo, H. E. , E. Becerra , R. Zalaquet , et al. 2016. “Atrial Function Assessed by Speckle Tracking Echocardiography Is a Good Predictor of Postoperative Atrial Fibrillation in Elderly Patients.” Echocardiography 33, no. 2: 242–248. 26394799 10.1111/echo.13059

[anec70230-bib-0047] Vieira, M. J. , R. Teixeira , L. Gonçalves , and B. J. Gersh . 2014. “Left Atrial Mechanics: Echocardiographic Assessment and Clinical Implications.” Journal of the American Society of Echocardiography 27, no. 5: 463–478. 24656882 10.1016/j.echo.2014.01.021

[anec70230-bib-0048] Wang, W.‐H. , S.‐H. Hsiao , K.‐L. Lin , C.‐J. Wu , P.‐L. Kang , and K.‐R. Chiou . 2012. “Left Atrial Expansion Index for Predicting Atrial Fibrillation and In‐Hospital Mortality After Coronary Artery Bypass Graft Surgery.” Annals of Thoracic Surgery 93, no. 3: 796–803. 22226234 10.1016/j.athoracsur.2011.11.007

[anec70230-bib-0049] Zacharias, A. , T. A. Schwann , C. J. Riordan , S. J. Durham , A. S. Shah , and R. H. Habib . 2005. “Obesity and Risk of New‐Onset Atrial Fibrillation After Cardiac Surgery.” Circulation 112, no. 21: 3247–3255. 16286585 10.1161/CIRCULATIONAHA.105.553743

[anec70230-bib-0050] Zhang, H. , H. Qiao , B. Yang , et al. 2023. “Development and Validation of a Diagnostic Model Based on Left Atrial Diameter to Predict Postoperative Atrial Fibrillation After Off‐Pump Coronary Artery Bypass Grafting.” Journal of Thoracic Disease 15, no. 7: 3708–3725. 37559620 10.21037/jtd-22-1706 PMC10407488

